# Editorial: Robotics software engineering

**DOI:** 10.3389/frobt.2025.1686496

**Published:** 2025-09-29

**Authors:** Federico Ciccozzi, Ivano Malavolta, Christopher Timperley, Andreas Angerer, Alwin Hoffmann

**Affiliations:** 1 Mälardalen University, Västerås, Sweden; 2 Vrije Universiteit Amsterdam, Amsterdam, Netherlands; 3 Carnegie Mellon University, Pittsburgh, United States; 4 XITASO Holding GmbH, Augsburg, Germany

**Keywords:** robotic software, robotic software architecture, robotic software development, robotic software framework, software testing, software engineering

## Introduction

Robotics software engineering stands at the intersection of multiple disciplines, where physical interaction with dynamic and uncertain environments amplifies the complexity of traditional software challenges. As robots become indispensable in domains such as manufacturing, healthcare, transportation, and exploration, they must exhibit high levels of autonomy, adaptability, robustness, and safety. Achieving these qualities requires not only technical breakthroughs in algorithms and hardware but also a strong foundation in software engineering principles tailored to the unique demands of robotics.

Robotics inherently involves multidisciplinary integration: navigation, motion planning, manipulation, perception, control, and human-robot interaction must all coalesce within a coherent software framework. Engineering these systems requires the careful coordination of experts from each domain, whose contributions must reliably interoperate, often in real time. Further challenges arise from operating in environments that are partially observable, dynamic, and sometimes adversarial, which raises the stakes for ensuring correctness, security, and resilience.

This Research Topic, *Robotics Software Engineering*, brings together a diverse Research Topic of contributions aimed at addressing foundational and emerging challenges in this space. Rather than presenting a simple catalog of articles, this editorial aims to situate these works within the broader themes that are shaping the future of robotics software.

## Bringing rigor to robotics: model-based engineering and formal methods

As robotic applications become increasingly safety-critical, ensuring correctness through formal verification becomes not just desirable but necessary. However formal methods remain difficult to apply due to the manual effort required to create models and extract system parameters. Dust et al. at Mälardalen University (Sweden) addressed this issue head-on with a model-driven methodology for the automated formal verification of ROS 2 systems. By integrating model transformation pipelines with real execution traces, this work demonstrates how verification can become more modular, reusable, and accessible to non-experts. This toolchain lowers the barrier to rigorous analysis, allowing developers to iteratively assess critical system properties such as timing and scheduling without being formal methods specialists.

Similarly, Barnett et al. (University of York, UK) proposed RoboArch, an architectural modeling language layered atop the formal DSL RoboChart, which advances the discipline by providing verifiable architectural abstractions. When applied in industrial contexts such as nuclear robotics, RoboArch emphasizes the value of model-driven design in bridging the gap between informal software practices and formal correctness in real-world systems.

## Architectures for adaptivity and reusability

Adaptation is a recurring theme in robotic systems, where conditions often change unpredictably. Several contributions explored adaptive software architectures as key enablers of robustness and long-term autonomy. ROSA, a knowledge-driven framework for robot self-adaptation proposed by Silva et al. (TU Delft, Netherlands), exemplifies this direction. It captures application-specific knowledge in structured models and reasons over them at runtime to guide both task execution and architectural configuration—a co-adaptation capability rarely addressed in robotics.

Complementing this, the survey on ontology-enabled autonomy by Aguado et al. (Universidad Politécnica de Madrid, Spain) examined how semantic knowledge and reasoning improve robot behavior in open-ended environments. By analyzing trends in the use of ontologies for fault recovery, mission planning, and behavior selection, the article highlights how structured, declarative knowledge can foster more explainable and dependable autonomy.

The contribution by Schneider et al. (Hochschule Bonn-Rhein-Sieg, Germany and KU Leuven, Belgium), Semantic Composition of Robotic Solver Algorithms, introduced a composable, graph-based methodology for algorithm synthesis. By leveraging standards from the Semantic Web, the authors enabled the reuse and symbolic generation of solver code across application domains, from kinematics to probabilistic inference. These developments advance the field toward software that not only adapts itself but also explains its logic, a key step for collaborative and trustworthy robots.

## Improving software quality through early validation and testing

Traditional debugging and validation approaches are inadequate for robotics, where errors discovered at runtime can lead to costly damage or unsafe behavior. Therefore, early and automated validation is crucial.

With EzSkiROS, Rizwan et al. (Lund University) and colleagues addressed this issue by using embedded domain-specific languages (DSLs) which enable the early detection of errors in robotic skill composition. By embedding checks in the design and deployment phases, this approach detected both high-level contract violations and low-level implementation bugs before they manifested during execution. This shift left in the validation pipeline shortens the debugging loop and improves overall safety.

At the other end of the deployment pipeline, with AAT4IRS, Dos Santos et al. (Université du Québec à Chicoutimi, Canada) introduced a novel framework for automated acceptance testing in industrial robotic systems. Built on behavior-driven development principles, this approach uses natural language to specify test scenarios, enabling cross-functional collaboration between engineers and stakeholders. Mutation testing results showed strong fault detection capability, indicating the practical utility of the framework in high-stakes industrial environments.

Simulation-based testing also receives attention. Despite its potential, it remains underused due to the complexity of scenario definition. To address this issue, the article by Ortega et al. (University of Bremen and Ruhr University Bochum, Germany) presented a composable scenario framework for testing mobile robots in virtual environments. By enabling developers to incrementally build and reuse complex scenarios, the approach reduces overhead while improving test coverage and configuration error detection.

## Foundations and infrastructure: languages, patterns, and performance

The infrastructure underlying robotic software must be efficient, reliable, and extensible. Several contributions examine foundational aspects, including runtime patterns, data structures, and energy consumption.

The study by Artigas et al. (KU Leuven and Flanders Make, Belgium) introduced software coordination patterns such as acquire-release and cache-awareness, alongside data structures such as Petri nets and finite state machines, to support real-time task execution. The proposed runtime infrastructure separates event firing from handling, facilitating distributed deployment and enabling consistent coordination across multiple robots.

The contribution by Albonico et al. (Federal University of Technology of Paraná, Brazil) and colleagues addressed an increasingly important concern—energy efficiency—by comparing the resource usage of ROS 2 nodes written in C++ and Python. Empirical results confirmed that C++ outperforms Python in energy consumption, particularly in high-frequency communication tasks, offering valuable guidance to developers who are optimizing for battery-powered or resource-constrained platforms.

Containerization also emerges as a promising strategy for scalable integration Cotugno et al. (Ocado Technology, UK) and colleagues proposed a containerized approach for multiform robotic architectures, demonstrating how virtualization can simplify the integration of third-party components without compromising performance. Evaluated in a real-world industrial robot, this method showed that modern software engineering practices such as containerization can be successfully adapted to robotics, reducing setup complexity while maintaining real-time guarantees.

## Toward a mature discipline of robotic software engineering

Taken together, the articles in this Research Topic reflect a field that is rapidly maturing—seeking not only functional solutions to robotic problems but principled, reusable, and verifiable engineering practices. From architectural modeling to energy-aware programming, from scenario-based testing to self-adaptive reasoning, each contribution addresses a facet of the broader challenge: how to engineer robotic systems that are not only intelligent, but also trustworthy, maintainable, and ready for real-world deployment.

This Research Topic fosters synergy between academia and industry, theoretical rigor and practical deployment. It invites the community to further explore the foundational questions of variability, modularity, reusability, validation, and automation in robotic software development. As robots increasingly share our spaces and tasks, the importance of sound engineering for their software only grows.

We hope these contributions inspire continued innovation and cross-disciplinary collaboration in the journey toward robust and dependable robotic systems.

